# Mainstreaming Local Food Species for Nutritional and Livelihood Security: Insights From Traditional Food Systems of *Adi* Community of Arunachal Pradesh, India

**DOI:** 10.3389/fnut.2021.590978

**Published:** 2021-08-16

**Authors:** Ranjay K. Singh, Rakesh Bhardwaj, Anamika Singh, Temin Payum, Arvind K. Rai, Anshuman Singh, Lobsang Wangchu, Sanjay Upadhyay

**Affiliations:** ^1^College of Horticulture and Forestry, Central Agricultural University, Pasighat, India; ^2^Banaras Hindu University, Varanasi, India; ^3^Department of Botany, JN College, Pasighat, India; ^4^ICAR-Central Soil Salinity Research Institute, Karnal, India

**Keywords:** wild edible plants, biocultural knowledge, nutritional values, ethnomedicine, recipe contest, food and nutritional policies, participatory learning

## Abstract

This study brings out the critical role of lesser-known local plant species in the food, nutrition and livelihood security of *Adi* community in Arunachal Pradesh, India. Considering women as a major custodian in knowledge and practices on foods, a total of 90 *Adi* women and 60 key knowledgeable community members (thus a total of 150 participants) were selected from East Siang and Upper Siang districts of Arunachal Pradesh. Data were collected using combination of methods including recipe contest, focus group discussion, personal interviews and laboratory analyses. The results indicated that *Adi* women were able to identify 39 bioculturally important species from a range of locally available plant species. Used alone or with other foods, these plants remain central to the *Adi* people's cultural identity and livelihood security. In addition to improving food and nutritional security, these species accessed from different land use systems, are also sold on the local markets to generate decent incomes. Of the species identified by *Adi* women, 28 were culturally shared and used frequently in food and ethnomedicine. Laboratory analyses of the selected 22 species revealed exceptionally high levels of minerals and other nutrients, such as proteins and anti-oxidants, supporting their traditional use for health benefits. Our study results provide valuable insights to the researchers to explore the vast hidden potential of these and other similar species for improving nutritional well-being of local communities in marginal areas. Adequate policy support is needed to enable *Adi* and other such marginalized communities to cope with challenges being posed to traditional food systems.

## Introduction

The social-ecological knowledge accumulated orally over generations has a great influence on traditional food systems and Indigenous biodiversity (1, 2). The Indigenous biodiversity, consisting of natural and domesticated local species nurtured and augmented over a certain geographical area (3), plays a pivotal role in the livelihood security of the local communities (4). There exists an intricate linkage between the foods consumed by Indigenous peoples and the local social-ecological conditions (5). Such locally available foods represent an easily accessible and affordable means to nutritional security (6, 7). The fact that women often play a critical role in sustaining and promoting the traditional foods, knowledge systems and livelihood security across the social-ecological systems is widely acknowledged (8–10). As biochemical constituents of locally consumed foods mostly remain unexplored, local people employ the traditional knowledge to understand the health benefits of different species used as food (11). Based on this knowledge, the local communities prioritize the conservation and management plans for local natural resources including agro-biodiversity (12). Of late, socio-economic and cultural shifts are increasingly altering the food preferences such that many traditional foods are being eclipsed by commercial foods (13), putting the traditional food systems and associated knowledge at risk (14). Conservation and promotion of local food practices are critical to global efforts toward safeguarding the culturally important foods and associated knowledge (1, 15).

Women of the tribal communities of Arunachal Pradesh (Ar P), India, are the real custodians of the local biodiversity (16) and traditional food systems (17, 18). They still rely on lesser-known local plant species for the household food and nutritional security (19). Many such species have exceptionally high levels of bio-active compounds displaying nutraceutical properties and contributing to overall health and well-being (20). However, their knowledge and participation are often little valued in the formal programs and policies aimed at strengthening family and community well-being (21). Therefore, validating this knowledge is particularly important (6, 22). The objectives of this study were: (i) to explore commonly used food plant species, and (ii) assess their nutritional, ethnomedicinal and cultural values identified by *Adi* women to better understand the intricate relationship with livelihoods.

## Conceptual Framework

Connecting cultural diversity with landscapes and ecosystems is critically important for sustaining the social-ecological resilience and traditional food systems of tribal and Indigenous peoples (23, 24). Understanding of such interactions can help to learn how a community has evolved its food system and add value to them. A better knowledge of such interactions is vital to learn the community's preferences in tune with livelihood needs and local conditions (7, 25, 26). In this study, we explored in collaboration with local *Adi* women, some of their locally valued food species that are still either semi-domesticated or collected from the wild. We also documented how the traditional knowledge embedded with these edible plants relates to their cultural significance, conservation, livelihood support and sustainability (8, 11, 27).

Indigenous and tribal communities of remote locations have evolved distinctive life-ways and cultural practices to meet their nutritional requirements (8, 28, 29). Drawing insights from Pretty (18), Kuhnlein et al. (6) and Kuhnlein (8), we tried to understand traditional ways and means employed by the *Adi* women to ensure nutritional and livelihood security. The complex interrelationships between food and culture of Indigenous people, like the *Adi*, are not readily apparent. Following Kuhnlein and Receveur (22), we enlisted the local food plants also being used as ethnomedicine by the *Adi*. The tribal communities, who are often marginalized, live in harsh ecological conditions (30), have evolved integrated strategies for accessing and using foods from diverse ecosystems (31). Following Kuhnlein (8), we documented some of these practices of *Adi* women relating to food and nutritional security that enrich the cultural diversity as well. We followed Shanley's approach (32) for devising a new methodology–the “recipe contest”–to mobilize the *Adi* community for exploring the plant food resources of high cultural and nutritional significance. This approach also helped us in rapport building with other stakeholders including community leaders and study participants. Drawing insights from Davidson-Hunt et al. (29), we collaborated with *Adi* women and leading knowledge holders over a long period (2006–2014) to gain deeper insights on current practices and concerns regarding traditional foods and to enhance the future opportunities. Through this approach, we were able to learn from *Adi* women as well as to support them through our research. For example, we undertook nutritional analyses of 22 important local food plant species. This was necessary to establish a scientific basis for the nutritional value of these species so that developmental agencies can devise appropriate future action plans for their multiple use and conservation. Our efforts in this direction led to positive results in terms of enhanced recognition of *Adi* women's traditional knowledge and linking these traditional foods with potential markets (33).

## Research Methodology

### The Study Area

Arunachal Pradesh (Ar P) is the largest state in northeastern India, covering a geographical area of 83, 743 km^2^. It lies between 26° 28′ to 29° 30′ N latitude and 91° 30′ to 97° 30′ E longitude. It has a hilly terrain, with mountains up to 7,090 m high. It is considered one of the most biodiverse regions of India, supporting extensive forests rich in plant and animal resources (34). There are 27 tribes and 110 ethnic groups in the state, most residing in close proximity to natural areas, on which they depend greatly for their livelihoods. The *Adi* are a major collective tribe living mainly in East Siang, Upper Siang, Upper and Lower Dibang Valley districts of Ar P (35). In both East Siang and Upper Siang (study districts), *jhum* cultivation (upland slash and burn agriculture) is a major socio-economic adaptive practice for *Adi*. Home gardens and community forests also provide considerable subsistence support to the *Adi*. In a nutshell, these integrated land use systems remain vital to food, nutritional and livelihood security of *Adis* (16, 18). Day-to-day interactions of *Adi* women with these local land use systems and resources over generations have shaped their distinct food preferences, practices and beliefs. While *Adi* women play a lead role in conserving and accessing the food resources for household livelihood security (16, 18), hunting of wild games and drudging tasks including slash and burn activities (*jhum* cultivation) are the major responsibilities of men.

### Sampling Design

#### Districts and Villages

Based on the people's ethnicity, relative dependency on natural resources and remoteness, three circles (administrative units) namely Pasighat and Mebo from East Siang and Mariyang from Upper Siang district were selected. Further, in consultation with Block Development Officers, we selected five villages randomly from each of these circles. Mirasm, Balek, Napit, Sibut and Yagrung villages were selected from Pasighat, while Ayeng, Kiyit, Borguli, Namsing and Sibuk from Mebo circle of East Siang district. Similarly, Mariyang H.Q, Damroh- Gingkong, Adi Pasi-Bine, Milang-Karket and Peki-Modi villages were selected from Mariyang circle of Upper Siang district.

#### Pilot Study and Duration: Key Insights for Methodology Enrichment

The studies involving human/animals were reviewed and approved by the Research Advisory Committee headed by the Dean, College of Horticulture and Forestry, Central Agricultural University, Pasighat, Arunachal Pradesh. Initially, we conducted a pilot study in two villages (December 2006 and January 2007) with six key knowledgeable women (3 from each village) after consultation with village *Gaon Bura* (GB) - customary chief and co-chiefs (Co-GB). The purpose was to generate key insights for the research methodology to be adopted for this study (36). Subsequently, different field activities and data collection exercises were carried out from 2008 to 2009. Building upon the knowledge of these activities, the initial plant samples were collected during August to October 2008 and 2009 for nutritional analysis. Further, a repeat sampling was done in 2012 and 2014 in the same months, to validate the results obtained in the previous analysis. Based on stability in results, data from these samplings were used as replicates in analysis.

#### Recipe Contests: Selection of Respondents

As recipe contest is considered a major participatory approach to record the women's knowledge and practices on food plants (9, 10), we organized recipe contests in each of the selected villages to record the diversity in traditional foods of plant and animal origin. Considering *Adi* women as more knowledgeable than men on food resources (16, 18), we requested them for free listing of food resources, with the objective of rapidly assessing food diversity and recording consensus among community members on shared cultural knowledge (37, 38). These contests were organized in each village, with the help of the village elders, *GB* and *Co*-*GB*- heading village *Kebang* (indigenous institution), and members of Village Panchayat (democratic institution). *Adi* women were informed about the recipe contests 15 days in advance. Interested women were invited to these recipe contests to demonstrate their traditional culinary skills at the village community hall (*mosup*) and to display dried and stored plant and animal samples used in the food preparation. The number of women participating in these contests varied from 20 to 35 (~28) per village. These women displayed 18–39 (~24) local plants used as ingredients in the traditional foods. The panel of judges for each contest consisted of a *GB* and Co-*GB*, two elderly women (who were not participants), and 2–3 (~2) research scientists. Thus, a total of 60 resource persons from 15 villages served as judges in the recipe contests and subsequent activities, as discussed later. The plant samples brought by the women contestants were collected, photographed and prepared as herbarium specimens for identification at the Botanical Survey of India (BSI) herbarium, Itanagar, Arunachal Pradesh. The author citations and botanical nomenclature were matched with IPNI checklist, PIC (Kew garden) of world flora, and identified by the name in current use (NCU) as per ICBN rules (St. Louis Code 2000) (39).

In each village, the traditional foods prepared and displayed by the *Adi* women during contests were evaluated by an expert panel using the Hedonic scale with slight modifications. The taste, flavor, texture, ethnicity, consumer preference, and overall community acceptance (38) were the scoring criteria for selecting the dishes and the contest winners. In case of any ambiguity in results, the decision of the *GB* and *Co-GB* (participating in the judge panel) was considered final. Each criterion was assigned a score between a maximum of “3” and a minimum of “0.” Thus, a particular food (using a local species) demonstrated by a woman could receive a maximum score of 18 and a minimum of 0. On the basis of mean values generated by the judges for each recipe and total number of foods presented by a woman, the overall recipe contest winners were identified. Six women from each study village (total 90) were conferred the awards in first, second, third and consolation (3 each) categories. These 90 women awardees were finally selected as the respondents of this study.

### Methods of Data Collection

#### Personal Interviews

A semi-structured interview schedule was developed with open-ended questions. The respondents were interviewed in the *Adi* dialect in presence of local guides (37). This interview schedule included questions on women's knowledge about local food plant species, association of plants with animal species, land use types where species are found, economic value of species, local creativity in mixing plants with different food resources and cultural and livelihood dimensions of the foods (Supplementary Material 1). The schedule was pilot tested with 5 *Adi* women in the non-sampled areas to assess the effectiveness of the language and suitability of the questions.

#### Participatory Techniques: Combined Approaches With Personal Interviews

A combined methodology is considered to be very effective in conducting in-depth interviews and discussions for collecting the knowledge (data) on traditional food practices (40). In addition to personal interviews, participant observation was applied as a participatory tool with knowledge holders to study some key food practices in the real field situations (41, 42). This exercise, under the guidance of local resource persons, helped us in recording the local practices and methods for collecting the food plants, food preparation and processing. As informal visits enrich the data by supplementing the participant observations on traditional food related knowledge and practices (43), we made several such visits during local festivals and cultural occasions (34 visits in total) to gain deeper insights into local food and cultural practices. Finally, random visits to the selected villages helped us in cross-checking and validating the local food-related knowledge documented during the previous visits/exercises. Transect walks (a participatory tool) are conducted in the presence of local knowledge holders to understand and verify the status and patterns of local resource use (44, 45). We conducted transect walks in each study village in consultation with village elders, *GB* and recipe contests winners to document the local plant and animal species used in the *Adi* foods. Focus group discussions were used to assess the preparation methodologies of certain dishes such as fermented foods and mixed foods (alcoholic beverages and plants mixed with fish and wild game).

#### Methods of Sampling and Nutritional Profiling of Selected Wild Vegetables

Based on the frequency of use, perceived nutritional importance, their shared cultural knowledge (37) and the high scores in recipe contests, 22 local food plant species were selected for their nutritional profiling. Samples were collected from study villages in each of four years (2008, 2009, 2012, and 2014), to account for variability in composition. For each species, about 2 kg of pooled sample was collected and composited. Each composite sample was analyzed in triplicate as analytical replicate to ensure repeatability and precision. ASFRM-6 (fish meal), ASFRM-14 (Rice flour) food reference standards obtained from Institute of Nutrition, Mahidol University, Thailand were used to ascertain recovery and accuracy of results. Results are presented as mean of means for four years data (*n* = 4) on per 100 gram fresh weight basis. The samples were processed using standard protocol and analyzed using official and standard methods. The moisture, ash, dietary fiber, protein, fat, starch and minerals contents were estimated using AOAC 934.01, AOAC 938.08, AOAC 985.29, AOAC 2001.11, AOAC 920.58, AOAC 996.11, and AOAC 999.11 methods (46), respectively. The total soluble sugars, ascorbic acid, total phenols and total flavonols were analyzed using Hedge and Hofreiter (47), Jagota and Dani (48), Singleton et al. (49), Quettier-Deleu et al. (50) methods, respectively. For details of analysis methods, see Supplementary Material 2.

#### Methods of Scoring the Selected Variables

To test the statistical significance of the correlation between land use type and access to different food species, we quantified these variables using a scoring technique: score of “1” was assigned to species conserved and accessed from *jhum* land (JL, based on topographical constraints); “2” to species from community forests (CF, based on limited conservation- allowing species to grow naturally) and “3” to those harvested from both JL and CF (cumulative weightage of both). Score “4” was assigned to the species accessed from both home gardens (HG) and JL, while a score “5” to those harvested from both HG and JL (cumulative weight of both). Seasonal availability of species was quantified by assigning a score of “1” to the species available for <3 months, “2” to those available for 3–6 months, “3” to availability for 6–9 months and “4” if available for >9 months. Frequencies of plant and animal species were accounted for analyzing diversity and similarity indices.

### Statistical Analysis

Data triangulation technique was applied to synthesize the information obtained through qualitative and quantitative approaches (51). Qualitative data, including socio-cultural, economic and ecological variables, were analyzed using thematic techniques (51). This data-set was complemented with verbatim responses recorded in our research diary to support the quantitative observations and explain the processes and interrelated dynamics of traditional food species. Nutritional values of local food plants presented as mean ± standard deviation. A “*t*”-test was used to assess the differences in incomes gained from the species accessed from different land use types using STAR software (52). Correlations between land use type and access to food species in a particular month/season were analyzed using Spearman rank correlation. Diversity and similarity analyses of the same set of data, and plant and animal species being used on cultural occasions, were computed using Shannon-Weaver index in the PAST statistical software (version 4, 2020) (53). Other quantitative data were entered into spread-sheet and analyzed using frequency and percentage. Our key findings were shared with the knowledge holders through a follow**-**up village meeting to clarify and refine the responses; especially in case of ambiguity or misinterpretation.

## Results

### Local Food Plant Resources

We recorded a total of 39 local plant species used by *Adi* women as traditional foods (Table 1). Out of these, 17 (43.59%) were domesticated and reared in different land use systems for food and cultural purposes. Fifteen (38.46%) were semi-domesticated (allowed to grow naturally), while 7 (17.95%) species were both semi-domesticated and domesticated. *Belang* (seeds of *Artocarpus heterophyllus*), *sirang* (*Castanopsis indica*), *taje* (*Amomum subulatum*), *angyat* (*Coix lacryma-jobi*) and *tasat* (*Arenga obtusifolia*) were the major food species consumed during droughts. Epidermal layer of *tasat*, though considered a delicacy among wealthier *Adi*, is also frequently consumed as bread during droughts by the poor. Selection of different parts of the local plants by *Adi* is mainly based on difference in taste in different seasons. For example, tender leaves of *ongin* (*Clerodendrum colebrookinanum*) are plucked during winter, while both older and tender leaves are chosen during rainy season.

**Table 1:** List of 39 local plant species used as food by *Adi* and their habitat and seasonal availability.

***Adi* name**	**Scientific name**	**Family**	**Habitat**	**Season of availability**	**Types of plant**	**Plant part, and mode of use**
*Aksap*	*Mussaenda roxburghii* Hook. f.	Rubiaceae	HG	September–November	SD	Leaf is boiled with other leafy vegetables
*Adi-ori*	*Eryngium foetidum* L.	Apiaceae	HG	(October–March)	D	Leaf is used as chutney, flavoring agent, and is boiled with meat, fish and other leafy vegetables
*Payin*	*Cucurbita moschata* Duchesne ex Poir.	Cucurbitaceae	HG	July–August	D	Tender leaves and flowers are used as vegetable and sometimes mixed with local fishes
*Takang*	*Diplazium esculentum* (Retz.) Sw.	Athyriaceae	HG	February–November	SD	Tender leaf is boiled with other leafy vegetables like *Spilanthes paniculata, Pouzolzia zeylanica, Fagopyrum esculentum*, etc.
*Angyat*	*Coix lacryma-jobi* L.	Poaceae	JL	October–December	D	Flour made from grain is used as food/beer preparation during lean period
*Bangko*	*Solanum spirale* Roxb.	Solanaceae	HG	August–November	D	Fruit is boiled with *Spilanthes paniculata, Zanthoxylum rhetsa* and *Allium hookerii* or with wet/dry fermented bamboo shoot. Tender leaves are used as vegetable
*Belang*	*Artocarpus heterophyllus* Lam.	Moraceae	JL	June–July	D	Ripen fruit is eaten; seed is boiled and eaten during lean period. Exchanged in barter also
*Buluka*	*Dendrocalamus giganteus* Wallich *ex* Munro.	Poaceae	JL and CF	September–October	SD + D	Culm-sheath of tender shoot is removed and tender shoot is chopped into fine size or cut into eatable size to boil as vegetable without fermentation
*Dibang*	*Bambusa tulda* Roxb.	Poaceae	CF	May–June	SD + D	Culm-sheath of tender shoot is removed and tender shoot is chopped or cut into eatable size (lengthwise) before cooking
*Dilap*	*Allium hookeri* Thwaites	Liliaceae	HG and JL	(November–February)	D	Bulb and leaf are used as flavoring agent, and cooked preferably with *Spilanthes paniculata, Zanthoxylum rhetsa* and *Solanum khasianum*.
*Eng*	*Dendrocalamus hamiltonii* Gamble	Poaceae	CF	September–October	SD + D	Culm-sheath of tender shoot is removed and tender shoot is chopped into fine size and kept in basket/bamboo container for fermentation or cut into eatable size to boil as vegetable without fermentation
*Engin*	*Dioscorea alata* L.	Dioscoreaceae	HG and JL	November–January	SD + D	Tuber is burnt under hot soil/boiled as food, basically used with tea as snack
*Gham-oying*	*Sauropus androgynus* (L) Merr.	Phyllanthaceae	HG and JL	Year round	D	Leaf is boiled with mixture of other leafy vegetables: *Spilanthes paniculata, Pouzolzia zeylanica, Fagopyrum esculentum*, etc.
*Gubor-oying*	*Amaranthus viridis* L.	Amaranthaceae	HG and JL	November–December	SD + D	Leaf is boiled with mixture of other leafy vegetables as above
*Hipe-oyik*	*Alternanthera philoxeroides* (Mart.) Griseb.	Amaranthaceae	HG	November–December	SD + D	Leaf is boiled with mixture of other leafy vegetables as above
*Kekir*	*Zingiber siangensis* Tatum and A K Das^*^	Zingiberaceae	HG	November–February	D	Used as major ingredients in many leafy vegetables. The rhizome, strongly aromatic and pungent, is a highly preferred local spice and taken while drinking local alcoholic beverages
*Kodum*	*Musa flaviflora* N. W. Simmonds	Musaceae	JL	September–October	SD	Ripe fruit is edible
*Koppi*	*Solanum khasianum* C. B Clarke	Solanaceae	HG	August–November	D	Fruit is boiled with *Spilanthes paniculata, Zanthoxylum rhetsa* and *Allium hookerii* or with wet/dry fermented bamboo shoot
*Kuso-belo*	*Ficus auriculala* Lour.	Moraceae	JL and CF	July–September	SD	Ripen fruit is eaten
*Lori*	*Piper pedicellatum* C. DC.	Piperaceae	HG	July–September	SD	Leaf is used as vegetable along with other leafy vegetables
*Marsang*	*Spilanthes paniculata* Wall. *ex* DC.	Asteraceae	HG	May–November	D	Leaf is steamed or boiled with other leafy vegetables like *Pouzolzia zeylanica, Clerodendrum colebrookinanum, Allium hookerii, Solanum kurzii, Solanaum torvum, Solanum khasianum, Gynura nepalensis*, etc.
*Mirung*	*Eleusine coracana* Gaertn.	Poaceae	JL	October–January	D	Powdered grain is used as food during lean periods and used in beer preparation during surplus periods
*Nemar*	*Piper mullesua* Buch. Ham. *ex* D. Don	Piparaceae	HG	September–December	SD	Steamed in bamboo with mixture of jungle meat and *Musa balbisiana* and *Musa flaviflora*
*Ongen*	*Gynura nepalensis* DC.	Asteraceae	HG and JL	September–December	D	Leaf is boiled with mixture of other leafy vegetables like *Spilanthes paniculata, Pouzolzia zeylanica, Fagopyrum esculentum*, etc.
*Oko-mamang*	*Solanum nigrum* L.	Solanaceae	HG and JL	July–November	D	Leaf is steamed with dry fermented bamboo shoot
*Okung*	*Fagopyrum esculentum* Moench.	Polygonaceae	HG and JL	March–November	D	Leaf is boiled with mixture of other leafy vegetables like *Spilanthes paniculata, Pouzolzia zeylanica, Fagopyrum esculentum*, etc.
*Onger*	*Zanthoxylum rhetsa* (Roxb.) DC.	Rutaceae	HG	August–October	SD	Leaf is steamed or boiled with other leafy/fruit vegetables such as *Pouzolzia zeylanica, Clerodendrum colebrookinanum, Gynura nepalensis, Allium hookerii, Solanaum torvum, Solanum khasianum*, etc.
*Ongin*	*Clerodendrum colebrookinanum* Walp.	Verbenaceae	HG	February–April	SD + D	Leaf is steamed or boiled preferably with other green leafy vegetables
*Oyik*	*Pouzolzia zeylanica* (L.) Benn. & R. Br.	Urticaceae	HG	October–November	SD	Leaf is boiled with fermented bamboo shoot and other leafy vegetables (*Pouzolzia zeylanica, Spilanthes paniculata, Zanthoxylum rhetsa*, etc.)
*Pakte*	*Musa balbisiana* Colla	Musaceae	HG and JL	Round the year	D	Ripe fruit is edible. Spadix is burned over fire and mixed with jungle meat (bird, squirrel, etc.). Leaves of *Piper pedicillatum* are steamed over fire and bamboo shoot is added to it
*Paput*	*Pseudognaphalium affine* (D. Don) Anderb.	Asteraceae	HG and JL	September–December	SD	Leaf is used as vegetable and mixed with other leafy vegetables
*Poi*	*Basella rubra* L.	Basellaceae	HG and JL	May–August	SD	Leaves consumed in boiled form and also mixed with meat and fish
*Sirang*	*Castanopsis indica* (Roxb. ex Lindl.) A. DC	Fagaceae	CF	October–December	SD	Epicarp is removed by lightly heating on pan over flame to get edible nut. Used as drought food also
*Tali*	*Amomum maximum* Roxb.	Zingiberaceae	JL	June–September	SD	Ripe fruit is plucked and epicarp is removed to eat mesocarp with seed (sweet in taste). Outer scape of young shoot is removed and inner tender scape is cooked with jungle meat/fish/leafy vegetables (*Pouzolzia zeylanica* and *Clerodendrum colebrookinanum*)
*Tapir*	*Phoebe cooperiana* P C Kanjilal and Das	Lauraceae	JL	August–October	SD	Epicarp and mesocarp are steamed in green bamboo before consumption
*Tare*	*Calamus erectus* Roxb.	Arecaceae	JL	May–July	SD	Epicarp is removed to eat mesocarp and nut
*Taje*	*Amomum subulatum* Roxb.	Zingiberaceae	HG and JL	April–August	D	Seeds are aromatic and pungent with pleasant taste. Raw part is boiled and used as vegetable after mixing with other leafy plants
*Tasat*	*Arenga obtusifolia* Griff.	Arecaceae	CF	October–March	SD	Epidermic layer is used as bread during drought, and also in making traditional alcoholic beverage *apong*
*Titabaigan*	*Solanum kurzii* Brace ex Prain	Solanaceae	HG	July–September	D	Fruits are taken as boiled vegetable, and mixed with a variety of fish and meat as well

* *It is treated as synonym of Z. officinale but many pharmaceutical experts mention that it is distinct*.

*HG, Home gardens; JL, Jhum lands; CF, Community forest*.

*Habitat data: D, Domesticated 17 species (43.59%); SD, Semi-domesticated but local 15 species (38.46%); D + SD, Domesticated and semi-domesticated 7 species (17.95%)*.

**Figure 1:**
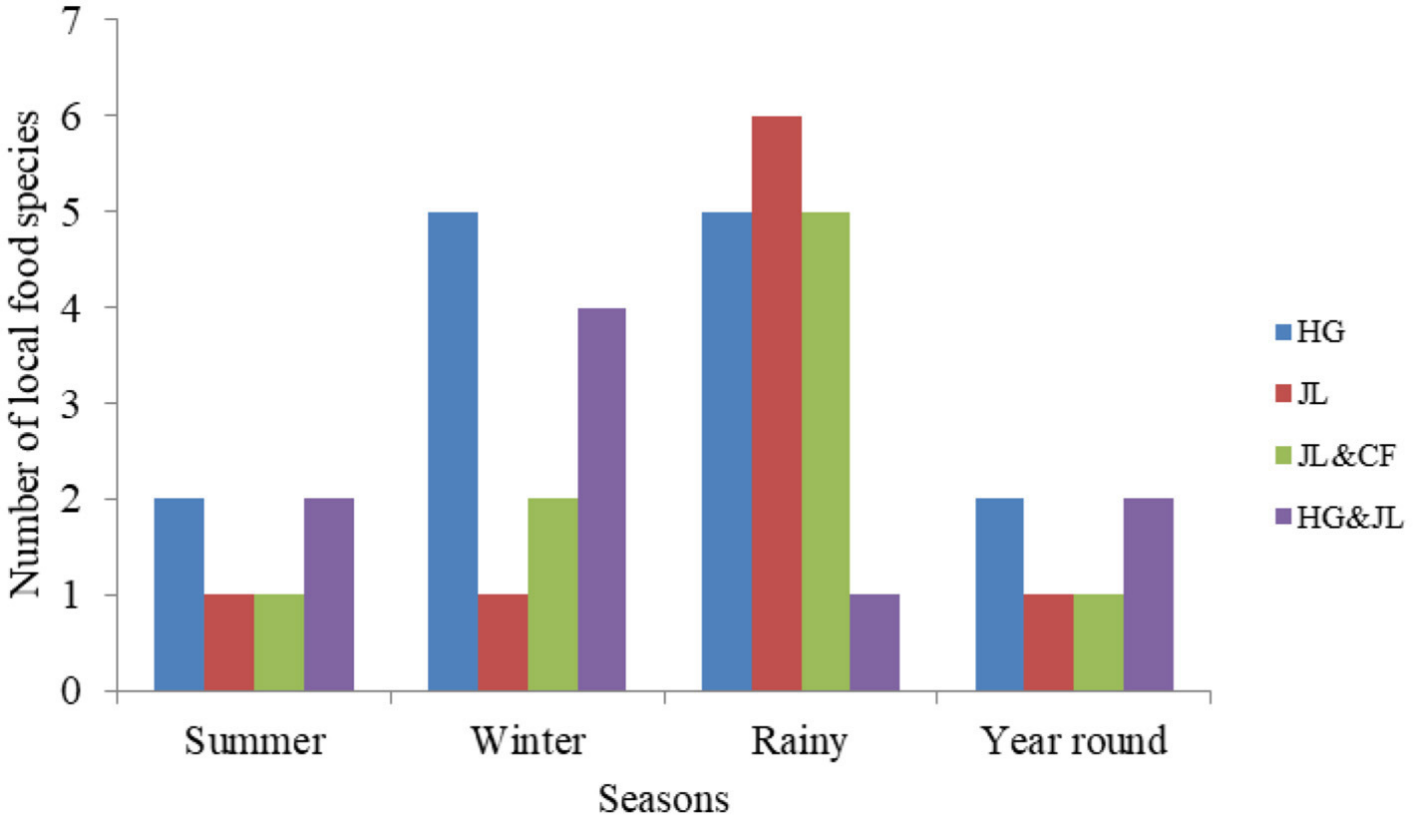
Seasonal availability of local food species from diverse land use types.

There was a considerable diversity (*H* = 3.44) in land use types for accessing food species by *Adi* women. Overall, a total of 17 species were accessed from these varied land use types (Figure 1) during the rainy season. Home gardens were found to be more prominent across the seasons for accessing food (14 species) followed by 9 species collected from the *jhum* lands. Another 9 species were from two land use systems combined i.e., *jhum* lands and community forests, or *jhum* lands and home gardens. There was an appreciable diversity (*H* = 3.32) in availability of seasonal food species, and *Adi* women were using 6 species round the year. Spearman rank correlation indicated a moderately significant correlation (*r* = 0. 524, *p*= 0.0004) between seasons and access to local food species from varied land use types.

### Income Generation From Local Food Plants

There were 39 local food plant species that helped generate subsistence income (Table 2). Community forests emerged as the major land use type, with mean income of INR 73.33 ± 19.45 per kg followed by *jhum* lands (INR 37.06 ± 4.09 per kg). With regard to income generation, there were significant differences between HG and JL (13 and 17 species, respectively; “*t*” = 2.39 *p* = 0.011) and between *jhum* lands and community forests (15 and 9 species, respectively; “*t*” = 2.72 *p* = 0.006) (Table 2). Despite a lower species count than other land use systems, food species conserved in community forests had a very high economic value.

**Table 2:** Income generated by the sale of local food plants species accessed from different land use systems.

**Land use types**	Income in INR/kg^*^	**Number of species**	**Comparison**	**“*t*” statistics**	***p*-value**
Home gardens (HG)	35.36 ± 4.12	13	HG vs. JL	2.39	0.011
*Jhum* lands (JL)	37.06 ± 4.09	17	HG vs. CF	0.75	0.230^NS^
Community forests (CF)	73.33 ± 19.45	9	JL vs. CF	2.72	0.006

* *The value with ± indicates standard error; NS, non-significant*.

### Ethnomedicinally Important Food Species

A total of 28 local food plant species were also considered important for ethno-medicinal value (Supplementary Table 1 in online resources 2). These were used for relief from various health problems and diseases including diabetes (*migom-koppi- Solanum torvum*), malaria (*bangko*- *Solanum spirale*), snake bites (*kekir- Zingiber siangensis*), high blood pressure (*ongin- Clerodendrum colebrookianum*) and fever (*nemar- Piper mullesua*), among others. Foods believed to improve the digestive system included *talap* (*Allium chinense*) and *kekir*. Foods made from unripe *koppi* and *kopi* (*Solanum kurzii*) were used as laxatives. Sometimes, a few species were mixed together with wild meat and fish to further improve their nutritional value. For example, *paput* (*Pseudognaphalium affine*)*, mamang* (*Physalis minima*), *sayong* (*Polygonum* sp.), and *onger* (*Zanthoxylum rhetsa*) were used as flavor-enhancers in meat dishes. Similarly, *nayang* (*Erigeron canadensis*), *tangum* (*Bidens pilosa*) and *gende* (*Gynura nepalensis*) were boiled together with wild game or local fish- *ngopi* (*Labeo dero* F. Hamilton) and given to the sick and lactating mothers as energy boosters.

Beliefs relating to food restrictions were also recorded, since they may aggravate particular aliments or disorders. For example, *Adi* women believed that lactating mothers should not consume *ongin* and *marshang* (*Spilanthes paniculata*) based foods, as their breast-fed babies may suffer from diarrhea and lactation may cease early (93.5 and 78.4%, response, respectively) (Supplementary Table 2 in online resources 2). Similarly, 76.9% of the respondents believed that consumption of bamboo shoots by a malaria patient was likely to further deteriorate his/her health condition.

### Nutritional Significance of Selected Local Food Plants

#### Leafy Vegetables

Among the key local food plants evaluated for nutritional composition, 16 were leafy vegetables (11 herbs and 5 shrubs) (Table 3A). Among these leafy vegetables, edible portions ranged from 51 to 92 %; minimum in *takang* (*Diplazium esculentum*) and maximum in *Adi-ori* (*Eryngium foetidum*). *Gham-oying* (*Sauropus androgynus*) had the lowest moisture content, but was highest in crude protein (9.14%), crude fat (3.69%), ash (3.31%), iron (27.3 mg/100 g), zinc (25.7 mg/100 g) and manganese (13.4 mg/100 g). It also had high content of phenols (591 mg/100 g gallic acid equivalent GAE), flavonols (67.7 mg/100 g quercetin equivalent QE), calcium (791 mg/100 g) and magnesium (72.3 mg/100 g) (Tables 3A,B).

**TABLE 3A:** Estimated values on various nutritional parameters of local food plants used by the *Adi*.

***Adi* name**	**Botanical name**	**Nutritional parameters^c^**							
	***Leafy vegetables***	**Edible %**	**Moisture %**	**Ash %**	**Crude fat %**	**Crude Protein %**	**Dietary fiber %**	**Total sugar %**	**Total starch %**
*Adi-ori*	*Eryngium foetidum* L.	92.5 ± 0.95	72.0 ± 1.56	0.87 ± 0.05	1.34 ± 0.20	2.77 ± 0.10	9.65 ± 0.27	0.84 ± 0.06	1.29 ± 0.06
*Akshap*	*Mussaenda roxburghii* Hook. f.	64.4 ± 9.24	84.7 ± 4.24	1.47 ± 0.47	1.34 ± 0.27	3.75 ± 0.14	10.3 ± 0.52	0.57 ± 0.12	1.19 ± 0.08
*Bangko*	*Solanum spirale* Roxb.	73.6 ± 3.1	76.3 ± 2.4	4.05 ± 0.25	0.93 ± 0.10	1.27 ± 0.10	8.84 ± 0.19	0.874 ± 0.09	2.13 ± 0.11
*Takanga*	*Diplazium esculentum* (Retz.) Sw.	51.4 ± 2.1	84.7 ± 1.5	1.91 ± 0.54	0.76 ± 0.10	4.79 ± 0.57	9.09 ± 0.17	0.941 ± 0.07	2.16 ± 0.12
*Dilap*	*Allium hookeri* Thwaites	89.3 ± 3.6	76.1 ± 2.1	3.96 0.26	0.83 ± 0.18	2.69 ± 0.11	10.6 ± 0.34	1.42 ± 0.08	3.01 ± 0.26
*Gham-oying*	*Sauropus androgynus* (L) Merr.	67.9 ± 5.1	71.4 ± 1.6	3.31 ± 0.16	3.69 ± 0.69	9.14 ± 0.90	9.20 ± 0.78	0.799 ± 0.06	2.51 ± 0.06
*Lori*	*Piper pedicellatum* C. DC.	74.9 ± 1.5	82.2 ± 1.6	2.74 ± 0.13	1.13 ± 0.75	4.14 ± 0.38	10.3 ± 2.36	0.831 ± 0.05	1.49 ± 0.17
*Marsang*	*Spilanthes paniculata* Wall. *ex* DC.	69.9 ± 10.9	85.9 ± 1.4	2.04 ± 0.16	1.25 ± 0.13	3.38 ± 0.58	6.22 ± 0.69	0.259 ± 0.02	0.93 ± 0.01
*Obul*	*Mackaya neesiana (*Wall.) Das	90.3 ± 1.1	85.7 ± 1.0	1.94 ± 0.05	0.79 ± 0.13	2.58 ± 0.16	6.13 ± 0.19	0.345 ± 0.03	1.89 ± 0.22
*Ongen* ^b^	*Gynura nepalensis* DC.	64.7 ± 5.6	82.4 ± 1.3	1.93 ± 0.14	1.68 ± 0.08	6.05 ± 0.73	7.45 ± 0.17	0.701 ± 0.03	1.25 ± 0.04
*Onger*	*Zanthoxylum rhetsa* (Roxb.) DC.	55.1 ± 6.0	81.8 ± 1.0	1.66 ± 0.24	1.96 ± 0.48	5.14 ± 0.36	7.97 ± 0.43	0.323 ± 0.04	0.55 ± 0.05
*Ongin*	*Clerodendrum colebrookinanum* Walp.	67.7 ± 6.6	79.6 ± 1.9	1.91 ± 0.12	1.67 ± 0.09	5.84 ± 1.11	8.46 ± 0.10	0.958 ± 0.07	1.14 ± 0.06
*Oyik*	*Pouzolzia zeylanica* (L.) Benn. & R. Br.	54.1 ± 1.8	79.0 ± 1.6	2.92 ± 0.24	1.05 ± 0.17	5.96 ± 0.08	9.92 ± 0.12	0.808 ± 0.07	0.95 ± 0.10
*Poi*	*Basella rubra* L.	75.3 ± 2.1	86.8 ± 1.5	2.60 ± 0.20	0.91 ± 0.05	4.75 ± 0.40	3.96 ± 0.28	0.862 ± 0.06	1.78 ± 0.09
*Payin*	*Cucurbita moschata* Duchesne ex Poir.	54.7 ± 1.5	82.6 ± 1.7	1.49 ± 0.13	1.09 ± 0.10	1.90 ± 0.14	8.08 ± 0.61	0.556 ± 0.03	1.12 ± 0.08
	Mean	69.7 ± 13	80.5 ± 4.9	2.37 ± 1.05	1.36 ± 0.74	4.24 ± 3.1	8.32 ± 1.9	0.739 ± 0.30	1.56 ± 0.67
	**Fruit based vegetables**								
*Kopir*	*Solanum indicum* L.	71.5 ± 1.4	73.0 ± 1.06	1.11 ± 0.67	2.09 ± 0.36	5.86 ± 0.47	15.6 ± 1.18	1.21 ± 0.09	1.73 ± 0.09
*Migom-koppi*	*Solanum torvum* Sw.	87.0 ± 2.7	77.3 ± 4.8	1.09 ± 0.47	1.18 ± 0.25	2.55 ± 0.53	14.4 ± 0.70	0.463 ± 0.08	1.24 ± 0.02
*Bangko*	*Solanum spirale* Roxb.	73.6 ± 4.4	80.0 ± 5.60	1.06 ± 0.06	1.89 ± 0.15	4.62 ± 0.37	8.77 ± 0.44	0.689 ± 0.11	1.31 ± 0.02
*Kopi*	*Solanum kurzii* Brace ex Prain	77.4 ± 4.7	76.5 ± 1.98	1.89 ± 0.62	1.52 ± 0.18	5.09 ± 1.07	6.89 ± 0.48	0.86 ± 0.13	2.31 ± 0.17
	Mean	77.4 ± 6.9	76.2 ± 3.1	1.79 ± 1.4	1.67 ± 0.40	4.53 ± 1.4	11.4 ± 4.2	0.806 ± 0.31	1.65 ± 0.49
	**Rhizomatous vegetables**
*Kekir*	*Zingiber siangensis* Tatum and A K Das	82.8 ± 1.4	80.7 ± 1.1	1.29 ± 0.14	2.36 ± 0.19	2.48 ± 0.34	12.2 ± 0.87	0.42 ± 0.08	0.97 ± 0.11
*Adi* ginger	*Zingiber officinale* Roscoe	83.4 ± 0.8	72.4 ± 1.3	0.50 ± 0.04	1.35 ± 0.15	2.86 ± 0.45	14.0 ± 0.14	1.46 ± 0.12	2.58 ± 0.21

*^a^ Also known as Dhekia saag*.

b *Known as gende also*.

c *Each composite sample was analyzed in triplicate. Results are presented as mean of means of four years data (n = 4), and mean value for each year is derived from three analytical replicates*.

**TABLE 3B:** Estimated values on various nutritional parameters of local food plants used by the *Adi*.

***Adi* name**	**Botanical name**	**Nutritional parameters^c^**										
		**Ascorbic acid**	**Phenol**	**Flavanol**	**Na**	**K**	**Ca**	**Mg**	**Fe**	**Zn**	**Mn**	**Co**
	**Leafy vegetables**	**mg/100 g**	**mg/100 g**	**mg/100 g**	**mg/100 g**	**mg/100 g**	**mg/100 g**	**mg/100 g**	**mg/100 g**	**mg/100 g**	**mg/100 g**	**μg/100 g**
*Adi-ori*	*Eryngium foetidum* L.	17.3 ± 0.97	130 ± 9.10	18.4 ± 0.92	78.6 ± 3.9	118 ± 8.3	143 ± 8.6	44.4 ± 2.9	8.47 ± 0.33	1.55 ± 0.09	2.61 ± 0.14	1.56 ± 0.26
*Aksap*	*Mussaenda roxburghii* Hook. f.	12.1 ± 0.29	383 ± 27	40.9 ± 2.9	33.7 ± 1.7	86.7 ± 6.1	476 ± 29	47.2 ± 1.9	3.79 ± 0.19	3.89 ± 0.23	4.53 ± 0.16	5.30 ± 0.42
*Bangko*	*Solanum spirale* Roxb.	23.2 ± 1.8	374 ± 23	38.7 ± 1.4	90.3 ± 2.7	476 ± 33	279 ± 11	62.5 ± 1.8	3.45 ± 0.78	1.22 ± 0.10	2.13 ± 0.11	3.62 ± 0.41
*Takang* ^a^	*Diplazium esculentum* (Retz.) Sw.	19.2 ± 1.6	213 ± 18	23.1 ± 1.3	35.2 ± 1.7	381 ± 27	67.4 ± 4.0	48.4 ± 2.0	8.33 ± 0.31	3.76 ± 0.31	2.17 ± 0.11	3.15 ± 0.33
*Dilap*	*Allium hookeri* Thwaites	23.1 ± 2.1	134 ± 17	48.2 ± 2.7	97.5 ± 3.2	270 ± 16	158 ± 22	33.3 ± 2.4	11.8 ± 0.48	0.716 ± 0.08	0.934 ± 0.10	3.84 ± 0.35
*Gham-oying*	*Sauropus androgynus* (L) Merr.	15.8 ± 2.6	591 ± 27	67.7 ± 1.3	131 ± 6.6	269 ± 19	791 ± 47	72.3 ± 1.4	27.3 ± 1.1	25.7 ± 1.5	13.4 ± 0.46	0.95 ± 0.17
*Lori*	*Piper pedicellatum* C. DC.	51.3 ± 1.9	385 ± 25	63.9 ± 7.2	54.0 ± 2.7	133 ± 9.3	293 ± 18	86.9 ± 1.7	10.3 ± 0.41	4.86 ± 0.29	2.10 ± 0.13	5.96 ± 0.51
*Marsang*	*Spilanthes paniculata* Wall. *ex* DC.	12.1 ± 0.9	185 ± 8.8	19.0 ± 1.03	52.2 ± 2.6	134 ± 9.3	281 ± 17	34.0 ± 1.7	2.26 ± 0.08	6.19 ± 0.37	1.68 ± 0.09	3.78 ± 0.28
*Obul*	*Mackaya neesiana (*Wall.) Das	17.5 ± 0.77	118 ± 20	20.8 ± 1.04	31.0 ± 1.5	90.4 ± 6.3	358 ± 22	44.9 ± 0.93	2.10 ± 0.11	1.39 ± 0.08	0.81 ± 0.12	3.52 ± 0.28
*Ongen* ^b^	*Gynura nepalensis* DC.	26.6 ± 2.5	338 ± 15	50.0 ± 2.1	472 ± 23	776 ± 54	456 ± 27	57.3 ± 1.1	5.31 ± 0.23	7.03 ± 0.42	6.73 ± 0.23	8.43 ± 0.67
*Onger*	*Zanthoxylum rhetsa* (Roxb.) DC.	14.8 ± 1.3	678 ± 18	41.8 ± 1.3	56.3 ± 2.8	129 ± 9.0	679 ± 41	45.2 ± 1.8	8.12 ± 0.32	6.67 ± 0.40	4.01 ± 0.11	7.36 ± 0.59
*Ongin*	*Clerodendrum colebrookinanum* Walp.	28.9 ± 2.3	214 ± 16	33.9 ± 1.7	185 ± 9.3	341 ± 23	513 ± 31	42.1 ± 1.7	6.35 ± 0.32	5.13 ± 0.33	3.92 ± 0.09	5.94 ± 0.51
*Oyik*	*Pouzolzia zeylanica* (L.) Benn. and R. Br.	18.0 ± 1.2	190 ± 8.0	33.0 ± 1.1	318 ± 21	514 ± 49	850 ± 69	60.6 ± 1.2	6.39 ± 0.29	3.53 ± 0.21	2.65 ± 0.13	7.60 ± 0.58
*Poi*	*Basella rubra* L.	81.0 ± 2.4	167 ± 12	26.4 ± 2.1	79.2 ± 3.9	192 ± 13	165 ± 9.9	198 ± 4.0	4.65 ± 0.24	1.64 ± 0.10	0.952 ± 0.03	1.46 ± 0.22
*Payin*	*Cucurbita moschata* Duchesne ex Poir.	31.80 ± 1.91	127 ± 5.8	35.6 ± 1.8	36.4 ± 1.8	412 ± 29	63.2 ± 3.8	52.4 ± 1.1	5.80 ± 0.23	2.34 ± 0.14	1.89 ± 0.06	4.54 ± 0.20
	Mean	26.2 ± 18	282 ± 173	37.4 ± 15	117 ± 124	288 ± 197	372 ± 252	62.0 ± 40	7.63 ± 6.1	5.04 ± 6.1	3.37 ± 3.2	4.47 ± 2.3
	**Fruit based vegetables**								
*Koppir*	*Solanum indicum* L.	20.0 ± 1.1	413 ± 31	67.5 ± 3.4	153 ± 7.7	323 ± 23	717 ± 43	69.8 ± 1.4	10.3 ± 0.41	5.12 ± 0.35	3.81 ± 0.11	11.9 ± 1.8
*Migom Koppi*	*Solanum torvum* Sw.	19.4 ± 0.72	221 ± 26	8.93 ± 1.1	88.5 ± 4.4	169 ± 12	92.4 ± 5.5	50.2 ± 1.0	4.73 ± 0.2	8.54 ± 1.9	1.67 ± 0.08	7.28 ± 0.63
*Bangko*	*Solanum spirale* Roxb.	26.70 ± 0.53	195 ± 18	53.7 ± 3.2	211 ± 11	357 ± 23	203 ± 12	33.1 ± 1.7	3.76 ± 0.15	3.29 ± 0.20	1.63 ± 0.05	3.76 ± 0.30
*Kopi*	*Solanum kurzii* Brace ex Prain	24.6 ± 0.48	336 ± 39	23.4 ± 3.7	56.1 ± 4.8	268 ± 18	137 ± 16	41.6 ± 2.8	3.55 ± 0.21	2.70 ± 0.15	0.912 ± 0.28	4.84 ± 0.33
	Mean	22.7 ± 3.5	291 ± 102	38.4 ± 27	127 ± 69	279 ± 82	287 ± 290	48.7 ± 16	5.59 ± 3.2	4.91 ± 2.6	2.01 ± 1.3	6.95 ± 3.6
	*Rhizomatous vegetables*	14.1 ± 0.71	306 ± 21	97.4 ± 6.8	58.3 ± 2.9	357 ± 25	103 ± 6.2	33.1 ± 2.7	3.76 ± 0.22	3.29 ± 0.50	0.632 ± 0.09	1.38 ± 0.17
*Kekir*	*Zingiber siangensis* Tatum and A K Das											
*Adi* ginger	*Zingiber officinale* Roscoe	12.6 ± 0.59	221 ± 9.2	78.3 ± 4.7	23.3 ± 1.2	368 ± 26	26.2 ± 1.6	74.8 ± 1.5	3.89 ± 0.23	1.15 ± 1.7	4.32 ± 0.14	1.26 ± 0.15

a *Also known as Dhekia saag*.

b *Known as gende also*.

c *Each composite sample was analyzed in triplicate. Results are presented as mean of means of four years data (n = 4), and mean value for each year is derived from three analytical replicates*.

*Na, Sodium, K, Potassium; Ca, Calcium; Mg, Magnesium; Fe, Iron; Zn, Zinc; Mn, Manganese; Co, Cobalt*.

The *piper* leaves displayed the highest dietary fiber (10.3%), ascorbic acid (51.3 mg/100 g) as well as high flavonol (63.9 mg/100 g QE) and ash (2.74%) content. However, piper leaves had moderate amounts of total phenols (385 mg/100 g GAE) (Tables 3A,B). Leaves of *dilap* had the highest ash content (3.96%), total sugars (1.42%), total starch (3.01%) and high iron (11.8 mg/100 g). *Onger* (*Zanthoxylum rhetsa*) leaves showed high amounts of crude fat (1.96%), total phenols (678 mg/100 g GAE) and calcium (679 mg/100 g). *Oyik* (*Pouzolzia zeylanica*) and *ongen* (*Gynura nepalensis*) were considered highly nutritious among the *Adi* peoples. An *Adi* proverb aptly signifies this perception:

“*Oyik doboname reyik reyik, ongen doboname regen regen*.”

[“Those who eat *oyik* are handsome and beautiful and those who eat *ogen* have good health and physique.” Terms *reyik-reyik* and *regen-regen* connote a healthy child].

Comparative assessment revealed that *ongen* was particularly rich in protein (6.05%), total ash (1.93%), crude fat (1.68) ascorbic acid (26.6 mg/100 g), phenols (338 mg/100 g) and flavanol (50.0 mg/100 g QE). It also displayed appreciable amounts of sodium (472 mg/100 g), potassium (776 mg/100 g), zinc (7.03 mg/100 g), manganese (6.73 mg/100 g) and cobalt (8.43 μg/100 g). Similarly, *oyik* had very high calcium content (850 mg/100 g), and was moderately rich in dietary fiber (9.92%), protein (5.96%), total ash (2.92%), sodium (318 mg/100 g), potassium (514 mg/100 g), and cobalt (7.60 μg/100 g) (Tables 3A,B).

Further, *poi* (*Basella rubra*) had the highest moisture (86.8%), ascorbic acid (81 mg/100 g) and magnesium (198 mg/100 g) levels. It also had moderate amounts of ash (2.60%) and starch (1.78%). Tender leaves of *payin* (*Cucurbita moschata*) showed below average values for most of the nutrients except for ascorbic acid, potassium and cobalt (Tables 3A,B). *Gham-oying* was found to be the best among all leafy vegetables in terms of several nutrients. Interestingly, local people also refer it as “multi-vitamin plant.”

#### Fruit Based Food

Results indicated that there were four Solanaceous fruit-based foods. These included *koppir* (*Solanum indicum*)*, migom-koppi* (*S. torvum*)*, bangko* (*S. spirale*) and *kopi* (*S. kurzii*). Despite low water content and lower levels of total sugars and starches, these four species had much higher amounts of other nutrients as compared to commonly consumed eggplant (*Solanum melongena)* fruit (protein 1.48%, ash 0.70%, fat 0.32%, dietary fiber 3.98%, and available carbohydrate 3.52%) Longvah et al. (54). *Koppir* was found to be higher in fat content (2.09%), protein (5.86%), dietary fiber (15.6%), total sugars (1.21%), total phenols (413 mg/100 g), and total flavanols (67.5 mg/100 g). This also displayed higher calcium (717 mg/100 g), magnesium (69.8 mg/100 g), iron (10.3 mg/100 g), manganese (3.81 mg/100 g), and cobalt (11.9 μg/100 g) as compared to the other three Solanaceous fruit vegetables.

#### Rhizome Based Food

Results revealed that there were two Zingiberale plants being used as traditional foods. In comparison to commonly cultivated ginger (*Z. officinale*), *kekir* (*Zingiber siangensis*) rhizome had a higher juice content, low sugars and starches. This species displayed a high content of ash, fat, total phenols, total flavanols, sodium, calcium and zinc.

### Cultural Dynamics of Local Food Plant Species

*Adi* women have developed traditional knowledge of local food plants over the course of time, demonstrating their culinary creativity in sustaining the cultural diversity. The following sections elaborate on the relationship between traditional knowledge of food and cultural diversity.

### Culture and Biodiversity: Intricate Relations

**Figure 2:**
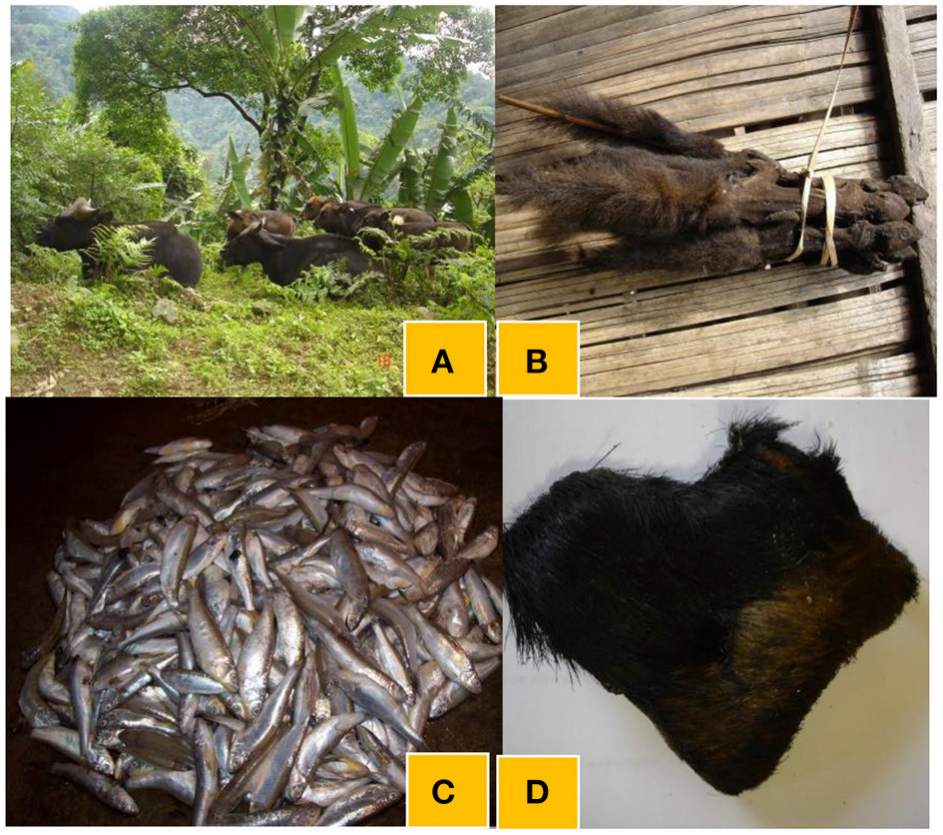
Local food plants and animal resources used in traditional foods of *Adi* community. **(A)** *Mithun* (*Bos frontalis*)- a culturally important semi-wild animal used in meat with plants; **(B)** *Kebung* (*Ratufa bicolor*)- a culturally important wild animal used for meat and gifted in festivals and marriages; **(C)** *Ngopi* fish (*Labeo dero*), used in fresh, fermented and dried form; **(D)** Dried deer meat. All the photos by Ranjay K Singh with consent from study participants.

**Figure 3:**
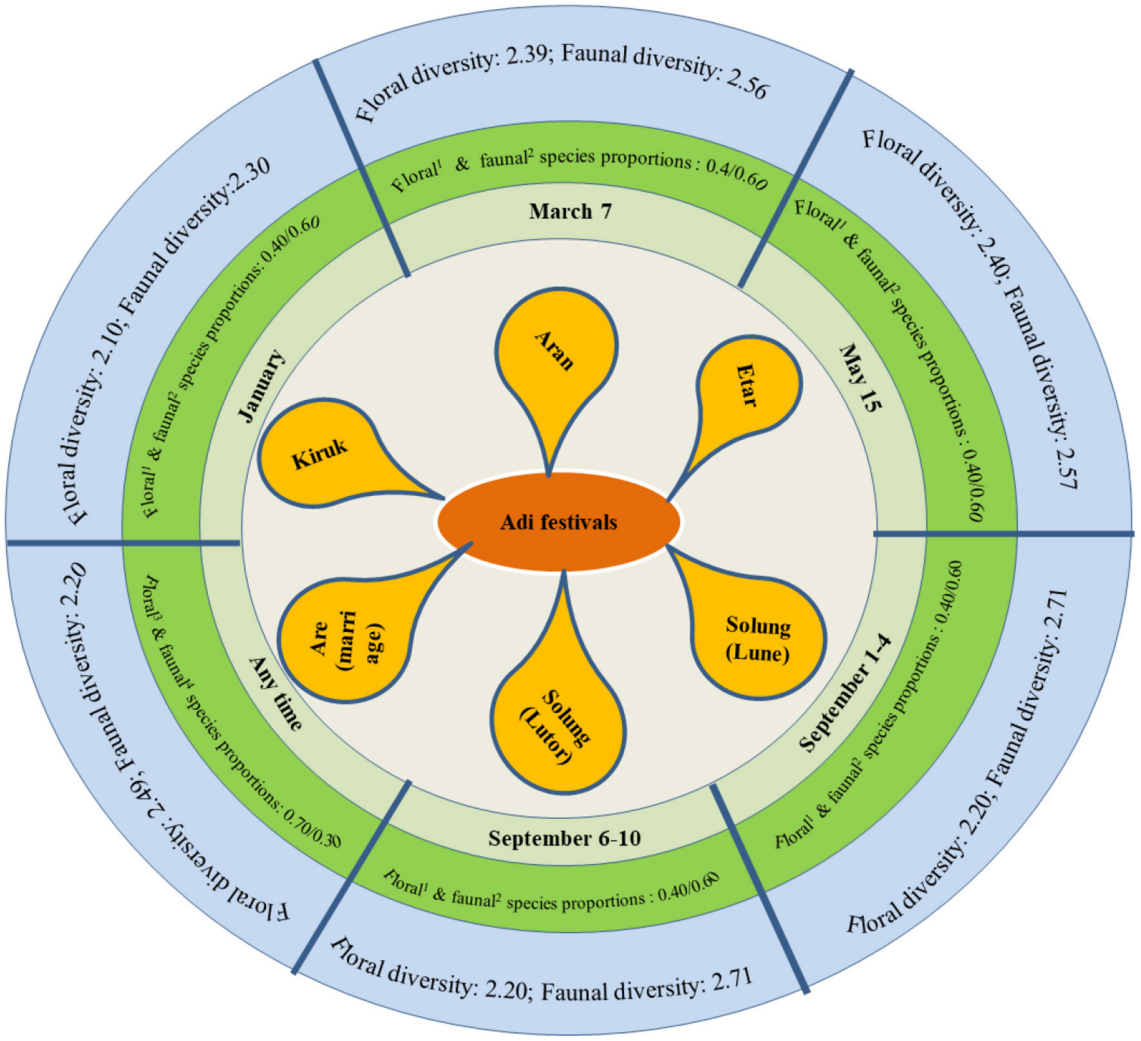
Major *Adi* festivals and their relations with local food plants and animal species as traditional foods. Diversity and similarity indices are based on Shannon-Weaver diversity analysis. ^1^Major local food plant species include: *kekir, takeng* (local ginger black in color), *onger, ongin, gam oying, koppi, koppir, paput, gende*, ikung and local paddy (*amkel, deku* and *jajung*) for food, and *apong* (fermented traditional beverage). The number of plant species may vary according to seasons. ^2^Major animal species used in food include: squirrels, *mithun*, wild rates, porcupine, deer, boar, local fishes, insects.

There were certain traditional foods prepared using locally available resources such as fish (e.g., *ngopi* and *tasum jhhinga machh*), insects (*tari-pug- Aspongopus najus*; *eri-pug*- *Samia cynthia*; citrus red ant-*Oecophylla smaragdina*, bamboo worm- *Omphisa fuscidentalis*, etc.) meat of *mithun* (*Bos frontalis*), chicken and wild animals. The *Adi* women uses leafy and other vegetables in conjunction with meat-based dishes such as fish, insect and wild games for enhanced taste and perceived health benefits. There are at least six major *Adi* festivals reflecting strong association between the culture and local dishes. Some local plants such as *amkel* rice*, kekir, takeng, onger, ongin, gham-oying, kopi, koppir, Adi-ori*, bamboo shoot*, namsing peron* are used as bulk ingredients in traditional foods along with foods prepared from domesticated animals (chicken and pork), fish, wild games [(*mithun, kebungs, nagopi* fish, deer (Figures 2A–D), boar, silkworm, bamboo worms*, tari-pug*, birds, etc.)] (Figure 3). During each *Adi* festival, celebrated in a particular month, these local food plants and meat/wild games are cooked together in different proportions (plant vs. animal: 60: 40) to make them delicious. Shannon-Weaver index revealed that despite changing proportions and combinations of food plants with animal species, considerable diversity was demonstrated through such cultural foods during the festivals. For example, during the *aran* festival, diverse local plants are cooked with such animals (Figure 3). Similarly, in other major festivals, such as *etor* and *solungs*, these foods are prepared and consumed. A team of *Adi* men and women collect dried wild games (dried rats, squirrel, *mithun* meat, etc.), rice grains, plant products (powder of *kopi, koppir, Adi*-*ori*) and fresh local vegetables from each household. Later on, these ingredients are cooked in the community hall (*mosup*), and are served along with cooked leafy vegetables to the community members, especially the elders, as a symbol of equitable sharing of the locally available nutritious food. These are supplemented by *apong*, a traditional (alcoholic) beverage prepared from *amkel* rice and or finger millet. During these celebrations, the *miris* (*Adi*'s cultural priest) perform a ritual dance and other *Adi* dancers sing folksongs signifying the cultural values of *jhum* lands, community forests, rivers and mountains.

### Local Creativity in Making Culturally Rich Foods

**Figure 4:**
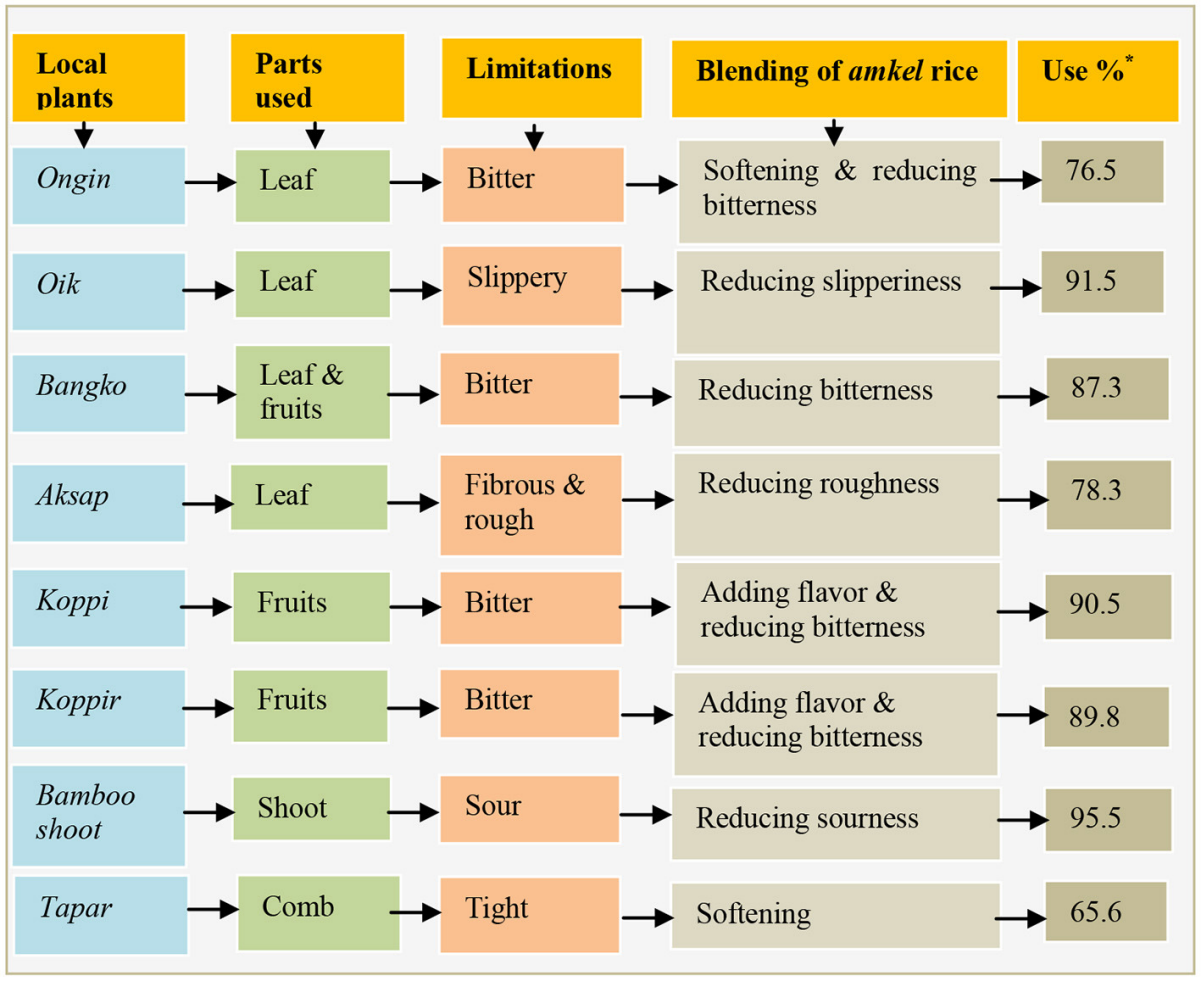
*Amkel* rice blended with some common ethnobotanicals to enhance food tastes. *Multiple percentage.

*Ongin, koppi, koppir* and *bangko* were the four most preferred local food plants by adults and elders, but were less preferred by children due to their “bitterness,” “roughness,” and “slippery” nature. To reduce the odd taste and enhance the softness, *Adi* women select tender parts of leaves or fruits and mix them with broken *amkel* rice (Figure 4). Similar practices were adopted for improving the palatability of “sour” plant foods. *Adi* women crush the soft corn grains between two stones to flatten the kernels. *Ngopi* fish is then boiled in water for 15–20 min, after which the bones are removed. The flesh is boiled again after being mixed with *luktir* (dried mixture of a local chili-*ritsar*, fruits of *bangko*, leaves of *onger* and dried bamboo shoots), along with salt and the flattened corn. The mixture is stirred well for 30 min, then water is added and is left to sit for a while. Thereafter, it is served with *namsing-peron* chutney made from fermented soybean (*Glycine max* L.) seeds, chili, ginger, garlic and salt.

Another food, called *khamti aamin/ambin*, is prepared with *amkel* rice soaked in cold water for 10 min and then made into a paste. Handful of *hilsha* (*Tenualosa ilisha* F. Hamilton) or the local fish (*ngopi*) are boiled (15 min) and deboned. Subsequently, the flesh is mixed with *amkel* rice paste together with salt, *chili, hopsi* (*Schizophyllum commune*) and *eepe* (dried powder of fermented bamboo shoots). The mixture is further cooked over a high heat for about 15–20 min, while being stirred continuously till it reaches to a ready-to-serve semi-liquid form. The fresh peel of *champa* fruits (*Dillenia indica* L.) are mixed with small fish like *ngopi*, to enhance the taste and keep the flesh firm during cooking.

Local beer (*apong*) is a specialty of *Adi* beverage prepared from time immemorial. Its quality mainly depends on the quality of rice grains used (*amkel* rice is most preferred) and on the yeast tablet (*siye*). *Adi* people make yeast tablets locally using some wild plants. These enhance the strength and storage quality of the beer. For this purpose, they use the green leaves of *rugzi* [*Pteridium aquilinum* (L.) Kuhn], *belang, rayil* [*Litsea cubeba* (Lour.) Pers.], *kopi* and tender shoots of sugarcane, blended together along with yeast culture from a previously fermented local beer lot.

## Discussion

### Food and Livelihood Security

The *Adis* live in remote locations of Ar P, and are recognized as marginalized in the context of India's developmental process (55). For these reasons, they have not had access to food grown in the plains and lowlands of India or to fully adopt scientific farming practices (56). Their very remoteness, however, has enabled them to evolve their own culturally rich food systems. This study demonstrated that *Adi* women are particularly knowledgeable about local plant species accessed from varied land use systems for food security. We documented a total of 39 local plant food species, used in dishes mixed with wild game. Gender-specific differences in food knowledge and practices have previously been reported (57). Accessing these food species provides a sustainable base for food and income to the *Adi* community. Indigenous tribal people living in remote locations adopt combined strategies and explore a variety of ecosystems in harvesting and preparing a large number of local species to ensure their nutritional security (58) and reduce the livelihood risks (59).

*Adi* women living in remote and fragile ecosystems face a set of interrelated food security and environmental challenges (60). They often attempt to solve these challenges by applying their knowledge holistically. For instance, an *Adi* woman might value her home garden and *jhum* land not just as a source of common herbs, but as a treasure trove of food, ethnomedicine and marketable products (61). *Adi* women's decisions about the use of a particular food might be affected by the knowledge acquired in past, perceived constraints on accessing it and the preference for local food habits (12). This was true with use of plants like *oyik* and *ogen* which were of high nutritional and cultural significance to *Adi*. The high calcium content along with other nutrients in *oyik* can be considered as a supportive evidence of their historical traditional knowledge; for example, in increasing the mothers' lactation (62). Therefore, these two local food species would have played important role in providing essential minerals (sodium, potassium, calcium, other nutrients as well as protein) given that milk and dairy product consumption is negligible among the *Adi*, and the common salt was scarce prior to the 1980s. Such relationships between a woman's choices with regard to food species, food habits and the reasoning behind use decisions, influenced by social-ecological factors (63), might not be easily discernible to an outsider but certainly affect the sustainability of local food resources (20, 41). As a result, it may undermine the potential future role of the local food species in meeting dietary diversity and food security (63). To address these issues, community-based prioritization and promotion of some potential local food plants through their enhancement, added to a scientific package of practices, can halt the erosion of food practices and associated biodiversity. Those concerned with ensuring people's food security and conserving local biodiversity (64) might investigate regions like Ar P, where high cultural diversity of foods is still evident (8, 65). Such regions and their local communities, if integrated, can have synergistic relations with policies contributing to better nutrition and health (e.g., Public Distribution System) and ecosystem integrity (66, 67).

### Nutritional and Health Security

Some of the local food species analyzed in our study were particularly nutritious. Globally, it is well-recognized that many local food plants consumed by the Indigenous peoples have high nutritional and medicinal qualities and have been selected based on years of informal experimentation (27). Local food species derived from various less manipulated land use systems, are typically higher in calcium, iron, magnesium, and vitamin C than their cultivated counterparts (68, 69). Other inquiries on nutritional composition of wild plants indicate a high content of proteins (70), fatty acids and amino acids (71, 72), and of minerals, especially K, Na, Ca, P, Mg (73, 74). Kuhnlein and Receveur (27) reported that many Indigenous foods, prepared from local species, are rich in protein, vitamins, iron, zinc, copper, magnesium and potassium (69). These local species play multiple roles not only in health promotion, but also in sustaining cultural diversity and knowledge. Our study also documented *Adi* women's use of 28 food plants in ethnomedicine. Their creativity in selecting, combining and processing local food/ethnomedicine species contributes to better health outcomes for the entire communities (1, 75). The use of certain foods as ethnomedicines may also help in addressing some contemporary health problems (76).

Some of the traditional food species documented here are particularly important during the lean seasons and adverse weather conditions (77). Some like *namdung* (*Perilla frutescens*) are recommended by elderly women to their younger pregnant counterparts as they are considered to be good for the health of the mother as well as the baby. An earlier study suggests that *namdung* is rich in omega 3 fatty acids, essential amino acids, manganese and zinc (71): nutrients needed more during pregnancy. Some other food plants, such as *onger, gham-oying, lori* and *ongen* (traditionally highly preferred) have also shown nutraceutical potential (phenols and flavanols). These observations support *Adi* women's traditional knowledge in selecting local food plants to maintain the health (78).

Meticulous processing and well-thought-out combinations of the local food resources by *Adi* women not only make them tasty and culturally unique, but also nutritionally balanced (22). Such practices, for example, were true with Solanaceous fruit vegetables having varying degrees of bitterness and less liked by younger people. With low water and carbohydrate levels, these species were found nutrient rich as compared to the common brinjal and were processed by women to make them acceptable to family members across the age groups. Surprisingly, several of these species are little known to outsiders and thus are not acknowledged in the research and policy arenas. Guijit et al. (25) referred to such food species as “hidden harvests,” and noted that they have the potential to enhance the overall well-being of marginalized communities. The *Adi* culture of using local food species, community-based cooking and sharing with others during cultural occasions are examples of equitability for those who seldom have access to such traditional foods (79). Significantly, most of the traditional food species (used alone or in combination with other plant and animal species) reported here are least known to other parts of India. In the remote and fragile ecosystems of Ar P where promotion of exotic food species is generally impractical, such species could potentially meet the current nutritional needs of marginalized people and thus enable them to adapt to environmental and livelihood challenges (69, 74).

## Conclusion and Policy Implications

*Adi* women of Arunachal Pradesh, India have rich knowledge on culturally and nutritionally important foods. Notably, this knowledge of nutritional biodiversity is also applied to maintain health and secure the subsistence livelihoods. *Jhum* lands and home gardens contribute significantly to food and nutrition security, together with community forests that provide species of high economic value. The *Adi* women adopt a meticulous strategy in using the local food plants with animals, fish and edible insects to fulfill their community's food and nutritional requirements. Many of the local food plant species (22) were nutritionally rich. In addition, 28 local plant species, including some from the 22 nutritionally assessed species, were also used simultaneously as ethnomedicines. Many of these species are rarely known to the rest of India. Some of them (22) are reported here for the first time for their nutritional value. These food resources (plants and animals) and related knowledge have a strong affinity with cultural diversity, Indigenous institutions and proximity to accessing local ecosystems. Based on the key findings of this study, the following points seem to be highly relevant to the future policy planning:

(i) Conservation plans for halting the genetic erosion of these valuable plant species need to be developed on priority. Further, package of practices for semi-domesticated and domesticated wild edible plants need to be developed to ensure their commercial production while ensuring sustainability and maintaining socio-ecological resilience of the traditional food systems.
(ii) Particular attention on the part of scientific and policy institutions is needed to create public awareness about the potential role and contributions of these traditional foods in health and nutrition, sustaining ecosystems and cultural knowledge.
(iii) Some of the plant-based foods with unique nutraceutical potential can be integrated with broader food and nutritional security programs (e.g., “Mid-Day-Meal” and PDS programs) in the study region and similar other geographical areas.
(iv) Several local vegetable species reported in this study (e.g., *oyik, ongen, gham-oying* and *dilap*) have high potential for introduction into similar other areas, as they would have high level of acceptance on account of taste and ease of integration with other foods.
(v) Local food plant resources can be mainstreamed by the state government to assist and support a “*bottom-up*” approach to development programs in promoting the better health, nutrition and women's empowerment. This can be pursued by leveraging the relevant provisions of the micro-entrepreneurship and skill development policies of India.

## Data Availability Statement

The original contributions presented in the study are included in the article/Supplementary Material, further inquiries can be directed to the corresponding authors.

## Ethics Statement

The studies involving human/animals were reviewed and approved by the Research Advisory Committee headed by the Dean, College of Horticulture and Forestry, Central Agricultural University, Pasighat, Arunachal Pradesh.

## Author Contributions

RS: substantial contributions to the conception and design of the work. RS, RB, TP, and AnaS: recipe contest and field-based activities, data collection. RB, AR, LW, and SU nutritional analysis and their interpretation. TP, SU, and AnsS: economic and cultural interpretation of relevant data. RS and RB: drafting the work and questions relating to results may be asked. RS and AnsS: revising it critically for important intellectual content. All authors final approval of the version to be published.

## Conflict of Interest

The authors declare that the research was conducted in the absence of any commercial or financial relationships that could be construed as a potential conflict of interest.

## Publisher's Note

All claims expressed in this article are solely those of the authors and do not necessarily represent those of their affiliated organizations, or those of the publisher, the editors and the reviewers. Any product that may be evaluated in this article, or claim that may be made by its manufacturer, is not guaranteed or endorsed by the publisher.
