# Supplementary material for: Mainstreaming Local Food Species for Nutritional and Livelihood Security: Insights From Traditional Food Systems of Adi Community of Arunachal Pradesh, India
Source: Front Nutr. 2021 Aug 16;8:590978. doi: 10.3389/fnut.2021.590978 (PMC8415219; doi:10.3389/fnut.2021.590978)
Supplement: Supplementary file 1 [file Data_Sheet_1.docx]

***Frontiers in Nutrition***

**Supplementary Material for Online Resources-1**

**Questionnaire Used For the Study**

***Mainstreaming Local Food Species for Nutritional and Livelihood Security: Insights from Traditional Food Systems of Adi Community of Arunachal Pradesh, India***

Ranjay K. Singh, Rakesh Bhardwaj, Anamika Singh, Temin Payum, Arvind K. Rai, Anshuman Singh, Lobsang Wangchu and Sanjay Upadhyay

**Questionnaire**

**A.** Personal profile

a Your name: b. Village: c. Age :

d**.** Land: i. *Jhum kheti*……. ii. Home garden iii. Community forest………iv. Others

e. Physical status: Non-pregnant/pregnant; Non-lactating / pregnant / lactating/ breastfed/breast fed/ complementary feed/ weaned/not applicable

**B.** Exploring the varieties of traditional plants and animal based food resources consumed

1. Name the types of food/animals you know and use

| Local name of species used in food items | Parts used | Habitats  (JL, HG, CF/others) | Seasonal availability | Effective availability period (months) | Domesticated/Semi-domesticated/others | Local economic value (Rs./kg) | Describe the relations with food & livelihood security |
| --- | --- | --- | --- | --- | --- | --- | --- |
| A. Cereals |  |  |  |  |  |  |  |
| 1 |  |  |  |  |  |  |  |
| 2 |  |  |  |  |  |  |  |
| 3 |  |  |  |  |  |  |  |
| 4 |  |  |  |  |  |  |  |
| 5 |  |  |  |  |  |  |  |
|  |  |  |  |  |  |  |  |
| B. Legumes |  |  |  |  |  |  |  |
| 1 |  |  |  |  |  |  |  |
| 2 |  |  |  |  |  |  |  |
| 3 |  |  |  |  |  |  |  |
| 4 |  |  |  |  |  |  |  |
| 5 |  |  |  |  |  |  |  |
|  |  |  |  |  |  |  |  |
| Local plants: C. Leaves |  |  |  |  |  |  |  |
| 1 |  |  |  |  |  |  |  |
| 2 |  |  |  |  |  |  |  |
| 3 |  |  |  |  |  |  |  |
| 4 |  |  |  |  |  |  |  |
| 5 |  |  |  |  |  |  |  |
| 6 |  |  |  |  |  |  |  |
| 7 |  |  |  |  |  |  |  |
| 8 |  |  |  |  |  |  |  |
|  |  |  |  |  |  |  |  |
| D.Tubers/rhizomes/  Bulbs  1 |  |  |  |  |  |  |  |
| 2 |  |  |  |  |  |  |  |
| 3 |  |  |  |  |  |  |  |
| 4 |  |  |  |  |  |  |  |
| 5 |  |  |  |  |  |  |  |
|  |  |  |  |  |  |  |  |
| E.Gourds/cucurbits |  |  |  |  |  |  |  |
| 1 |  |  |  |  |  |  |  |
| 2 |  |  |  |  |  |  |  |
| 3 |  |  |  |  |  |  |  |
| 4 |  |  |  |  |  |  |  |
|  |  |  |  |  |  |  |  |
| F.Fruits |  |  |  |  |  |  |  |
| 1 |  |  |  |  |  |  |  |
| 2 |  |  |  |  |  |  |  |
| 3 |  |  |  |  |  |  |  |
| 4 |  |  |  |  |  |  |  |
|  |  |  |  |  |  |  |  |
| G. Other plants not covered above |  |  |  |  |  |  |  |
| 1 |  |  |  |  |  |  |  |
| 2 |  |  |  |  |  |  |  |
| 3 |  |  |  |  |  |  |  |
|  |  |  |  |  |  |  |  |
| H. Name of meat/wild games |  |  |  |  |  |  |  |
| 1 |  |  |  |  |  |  |  |
| 2 |  |  |  |  |  |  |  |
| 3 |  |  |  |  |  |  |  |
| 4 |  |  |  |  |  |  |  |
| 5 |  |  |  |  |  |  |  |
| 6 |  |  |  |  |  |  |  |
|  |  |  |  |  |  |  |  |
| I. Use of dried meat/animals |  |  |  |  |  |  |  |
| 1 |  |  |  |  |  |  |  |
| 2 |  |  |  |  |  |  |  |
| 3 |  |  |  |  |  |  |  |
| 4 |  |  |  |  |  |  |  |
| 5 |  |  |  |  |  |  |  |
| 6 |  |  |  |  |  |  |  |
| 7 |  |  |  |  |  |  |  |
|  |  |  |  |  |  |  |  |

JL= *Jhum* land; HG=Hoe garden; CF= Community forest

2.Please explain how you use different plants and animals resources in your food systems?

| Sr N. | Name of food plants | Name of animal resources | Ratio of use | From where accessed (land use types) | Specific method of preparation | Do you conserve intentionally and or nurtured naturally | Additional information |
| --- | --- | --- | --- | --- | --- | --- | --- |
| 1 |  |  |  |  |  |  |  |
| 2 |  |  |  |  |  |  |  |
| 3 |  |  |  |  |  |  |  |
| 4 |  |  |  |  |  |  |  |
| 5 |  |  |  |  |  |  |  |
| 6 |  |  |  |  |  |  |  |
| 7 |  |  |  |  |  |  |  |
|  |  |  |  |  |  |  |  |

1. Do women, children and men eat the same foods? Or does it vary by age?. If it varies, then please describe your own creativity how you manipulate/mix or blend different plants and animal resources to make it consumable to a particular group?

a

b

c

d

1. Please narrate about the list of fermented foods-if any, and their use/supplement with various plant and animal based foods?

| Sr. N. | Name of the fermented foods | Supplemented with | Preparation methods | Additional information | Add Focus Group Discussion to go in-depth |
| --- | --- | --- | --- | --- | --- |
| 2 |  |  |  |  |  |
| 3 |  |  |  |  |  |
| 4 |  |  |  |  |  |
| 5 |  |  |  |  |  |
| 6 |  |  |  |  |  |
| 7 |  |  |  |  |  |

**C.** Perception ion of *Adi* women about the nutritional and therapeutic aspects of traditional foods

1. Please name and describe the local plant species you perceive nutritionally and ethno-medicinally valuable?

| Sr. N. | Name of plant species | Perceived nutritional value | Perceived ethnomedicinal values | Part(s) used | Any specific method of preparation | Relation with food and livelihood security | Additional information |
| --- | --- | --- | --- | --- | --- | --- | --- |
| 2 |  |  |  |  |  |  |  |
| 3 |  |  |  |  |  |  |  |
| 4 |  |  |  |  |  |  |  |
| 5 |  |  |  |  |  |  |  |
| 6 |  |  |  |  |  |  |  |
| 7 |  |  |  |  |  |  |  |

2. Do you perceive that your ethnic foods are better over the other available modern foods? If yes, then what are those aspects?

a

b

c

d

e

f

**D.** Cultural dimensions of foods

2. Name the foods which are specifically used on particular occasions like festival, marriage, etc.:

| Sr. N. | Name of the food | Name of cultural occasion | Use proportion (major or minor) | Your local view about hoe it help in conservation of species | To be learned with participant observations |
| --- | --- | --- | --- | --- | --- |
| 2 |  |  |  |  |  |
| 3 |  |  |  |  |  |
| 4 |  |  |  |  |  |
| 5 |  |  |  |  |  |
| 6 |  |  |  |  |  |
| 7 |  |  |  |  |  |

3. Name of foods which are associated with certain taboos, beliefs or spiritual values?

A

b

c

d

e

4.The traditional foods you try to avoid during the following health problems

| Sr. N. | Health problems | Name of the foods | Reasons | Additional information |
| --- | --- | --- | --- | --- |
| 2 | Pregnancy |  |  |  |
| 3 | Lactation |  |  |  |
| 4 | Diarrhoea |  |  |  |
| 5 | Jaundice |  |  |  |
| 6 | Malaria |  |  |  |
| 7 | Diabetes |  |  |  |
| 8 | High blood pressure |  |  |  |
| 9 | Others |  |  |  |

**E.** Historical, climatic and change context of foods

1. Please name the foods which were available earlier about 20 to 30 years back, but now used least?

| Sr. N. | Name of the food plants | Nam of the animals | Place of availability | Season |
| --- | --- | --- | --- | --- |
| 2 |  |  |  |  |
| 3 |  |  |  |  |
| 4 |  |  |  |  |
| 5 |  |  |  |  |
| 6 |  |  |  |  |
| 7 |  |  |  |  |
| 8 |  |  |  |  |
|  |  |  |  |  |

2. Name those foods known to be used during periods of drought, epidemic and flood?

| Sr. N. | Name of resources | Drought/epidemic/flood | Place of availability | Reason of use |
| --- | --- | --- | --- | --- |
| 2 |  |  |  |  |
| 3 |  |  |  |  |
| 4 |  |  |  |  |
| 5 |  |  |  |  |
| 6 |  |  |  |  |
| 7 |  |  |  |  |
| 8 |  |  |  |  |
|  |  |  |  |  |

3. Please provide information about plant and animal based food resources you want to share but are not captured in any of the above questions asked _______________________

__________________________________________________________________
