# Supplementary material for: Mainstreaming Local Food Species for Nutritional and Livelihood Security: Insights From Traditional Food Systems of Adi Community of Arunachal Pradesh, India
Source: Front Nutr. 2021 Aug 16;8:590978. doi: 10.3389/fnut.2021.590978 (PMC8415219; doi:10.3389/fnut.2021.590978)
Supplement: Supplementary file 2 [file Data_Sheet_2.docx]

***Frontiers in Nutrition***

**Supplementary Material for Online Resources-2**

***Mainstreaming Local Food Species for Nutritional and Livelihood Security: Insights from Traditional Food Systems of Adi Community of Arunachal Pradesh, India***

Ranjay K. Singh, Rakesh Bhardwaj, Anamika Singh, Temin Payum, Arvind K. Rai, Anshuman Singh, Lobsang Wangchu and Sanjay Upadhyay

1. **Methodology**

**1.DETAILED METHDOLOGY OF NUTRITIONAL PROFILING OF SELECTED 22 WILD FOOD PLANTS**

Based on participatory recipe contests, 22 local food plants were prioritized for nutritional profiling based on their common usage in food and nutrition, scores assigned to each during recipe contest and non-availability of nutrient composition data.

**1.1 SAMPLING AND PREPARATION OF FOOD SPECIES**

Samples were collected over the years (2008, 2009, 2012 and 2014) from study villages to account for variability in composition. For each plant species used as traditional food, about 2 kg of pooled sample was collected. Samples were brought to laboratory in refrigerated insulated boxes within twelve hours from harvesting. On arrival samples were assigned sample number, laboratory entry number, date and place of sample collection. Each sample was first rinsed with tap water to remove adhering dirt followed by de-ionised water, and then kept under fan for one hour to remove water from surface. Edible and non-edible portions were separated with the help of study respondents, each time different respondents were involved to account for individual variation in choosing edible portions. Percentage of edible and non-edible portions was calculated as average.

Edible portion were chopped using stainless steel knives, there after shade dried initially for six hours under the fan and then dried in oven at 60 ± 0.5^0^C for twenty four hours or more till constant weight. Dried samples were grinded using stainless steel dry grinder. Grinded samples were sieved through test sieve of MICS – 400, to obtain homogeneity of samples. Homogenized samples were kept in polypropylene air tight sample containers and stored under dark in desiccators. All analysis was completed within three weeks of harvesting and samples were analyzed in triplicate. Results are presented as mean value on per 100 gram fresh weight basis.

**1.2.** **PROXIMATE COMPONENT ESTIMATION OF SELECTED SPECIES**

The proximate composition for 22 plant species was determined using the official methods (AOAC, 2005) (1) viz. moisture (AOAC 934.01), ash (AOAC 938.08), dietary fiber (AOAC 985.29), protein (AOAC 2001.11) and fat (AOAC 920.58). Sample preparation for total sugar, total starch, total phenols and total flavanols was done as described by Arivalagan et al. (2). Briefly, 100 mg of dried and homogenized samples were extracted thrice with 5 ml of 80% (v/v) ethanolat 60^0^ C for 60 min. After each extraction, samples were centrifuged at 5000g for 15 min and supernatant was pooled. Extract was dried in boiling water bath, then dissolved in water for estimation of sugar, phenols and flavonols while the residue left over of sample from 80% ethanol extraction was used for starch estimation. Total soluble sugar content in the extract was determined using anthrone reagent method (3) and starch content was estimated as per AOAC 996.11. Determination of TPC in the extract was done using Folin-Ciocalteu (FC) assay as described by Singleton et al. (4) with slight modifications. The results were expressed as gallic acid equivalent (GAE). Total flavonol were estimated by aluminium chloride spectrophometric method as described by Quettier-Deleu et al. (5) and results were expressed as Quercetin equivalent (QE).

Minerals profiling was done as per AOAC 999.11 using PerkinElmer AAnalyst 200 atomic absorption spectrometer. ASFRM-6 (fish meal), ASFRM-14 (Rice flour) food reference standards obtained from Institute of Nutrition, Mahidol University, Thailand were used to ascertain recovery and validate analytical methods.

**REFERENCES**

1. Association of Official Analytical Chemists, 2005. Official Method 999.11: Determination of Lead, Cadmium, Copper, Iron, and Zinc in Foods.
2. Arivalagan M, Roy TK, Yasmeen AM, Pavithra, KC, Jwala PN, Shivasankara KS, Manikantan MR, Hebbar KB, Kanade SR. 2018. Extraction of phenolic compounds with antioxidant potential from coconut (Cocos nucifera L.) testa and identification of phenolic acids and flavonoids using UPLC coupled with TQD-MS/MS, LWT. *Food Sci & Tech.* doi: 10.1016/j.lwt.2018.02.024.
3. Hedge, J E and Hofreiter, B T (1962) In: Carbohydrate Chemistry **17** (Eds Whistler R L and Be Miller, J N) Academic Press New York.
4. Singleton, V.L., Orthofer, R. and Lamuela-Raventós, R.M., 1999. [14] Analysis of total phenols and other oxidation substrates and antioxidants by means of folin-ciocalteu reagent. In *Methods in Enzymy* (299): 152-178. Academic press.
5. Quettier-Deleu, C., Gressier, B., Vasseur, J., Dine, T., Brunet, C., Luyckx, M., Cazin, M., Cazin, J.C., Bailleul, F. and Trotin, F., 2000. Phenolic compounds and antioxidant activities of buckwheat (Fagopyrum esculentum Moench) hulls and flour. *J Ethnopharm*, 72(1-2): 35-42.

**2. RESULTS**

- 1. **FOOD PLANTS USED AS ETHNOMEDICINES**

**TABLE 1** Food plants ( 28) used as ethnomedicines by *Adi* women.

| Local Name | Scientific name | Parts used is food | Parts/form used in medicine | Medicinal values as perceived by *Adi* women |
| --- | --- | --- | --- | --- |
| *Adi-ada* | *Zingiber officinale*  Roscoe | Rhizome | Rhizome | Used in skin diseases and cough and fever |
| *Akshap* | *Mussaenda roxburghii* Hook.f. | Leaf | Leaf | Improving eye site |
| *Angyat* | *Paspalum scrobiculatum* L*.* | Seeds | Fermented seeds | Energy enhancer and treat waist pain after delivery |
| *Bangko* | *Solanum spirale* Roxb. | Leaf | Fruits and roots | Reduce diabetes and used during malaria |
| *Bhoot jolakia* | *Capsicum Chinense* x C. frutescens | Fruits | Leaf and fruits | Treating malaria |
| *Champa* | *Dillenia indica* L. | Fruits used with fish and | Fruits | Controlling stomach pain |
| *Choulai* | *Amaranthus viridis* L. | Leaf | Leaf | Keeping pregnant women healthy |
| *Dilap* | *Allium hookeri* Thwaites | Entire plant | Bulbs | Remedy in snakes bite |
| *Gende* | *Gynura nepalensis* DC. | Tender stem and leaf | Leaf | Keeping pregnant women and sick persons healthy |
| *Goba- oying* | *Amaranthus spinosus* L. | Leaf | Leaf and seeds both | Blood enhancer among old persons and pregnant women |
| *Jipin* | *Citrus medica* L. |  | Fermented fruits | Treating jaundice and stomach disorders |
| *Kekir* | *Zingiber siangensis* Tatum & A K Das^*^ | Rhizome | Rhizome | Cough, fever, diabetes, stomach pain and snake bite |
| *Mogom-koppi* | *Solanum torvum* Sw. | Fruits | Fruits | Treating diabetes |
| *Koppir* | *Solanum khasianum* C.B Clarke | Fruits | Fruits | Used as laxative and treating diabetes |
| *Kordoe* | *Averrhoa carambola* L. | Fruits | Leaf and fruits | Curing jaundice |
| *Lai saag* | *Brassica juncea* var. rugosa | Leaf | Leaf | Used as vitamin and minerals rich leafy vegetable, especially for old aged and pregnant women |
| *Marsang* | *Spilanthes paniculata* Wall. *ex* DC. | Leaf | Leaf with flowers | Helps in reducing the diarrhea |
| *Namdung* | *Perilla frutescens* (L.) Britton | Seeds | Seeds | Given more the pregnant women for keeping them and fetus healthy |
| *Nemar* | *Piper mullesua*Buch. Ham.*ex*D. Don | Fruits | Dried fruits | Treating cough and fever |
| *Ogjok* | *Bauhinia variegata* L. | Leaf | Flowers | Used in dysentery and stomach pain |
| *Omri* | *Carica papaya* L. | Unripe fruits | Unripe fruits | Lactating mother, laxative |
| *Ongen* | *Gynura nepalensis* DC. | Leaf |  | Improving eye site |
| *Onger* | *Zanthoxylum rhetsa* (Roxb.) DC. | Leaf | Tender leaves | Avoiding tapeworms due to frequent use of pork and other stomach disorders |
| *Ongin* | *Clerodendrum colebrookinanum* Walp. | Leaf | Tender leaves | Reduce the high blood pressure |
| *Oyik* | *Pouzolzia zeylanica* (L.) Benn. & R. Br. | Leaf | Leaf | Improving eye site |
| *Paput* | *Pseudognaphalium affine* (D.Don) Anderb. | Leaf | Leaf | Improving blood percent |
| *Rouri* | *Piper peepuloides* Roxb. | Leaf |  | Improving blood percent |
| *Talap* | *Allium chinense* G.Don | Bulb | Tender bulb and leaves | Improving digestion |

*It is treated as synonym of *Z. officinale* but many pharmaceutical experts maintain that it is distinct

**1.2 HEALTH RELATED BELIEF OF *ADI* COMMUNITY**

**TABLE 2** Health related belief associated with traditional foods of *Adi* community.

| Problems | Name of food | Reason for avoidance quoted by users | Response%* |
| --- | --- | --- | --- |
| Pregnancy | *Kochu* (colocassia) leaf | Sometimes, it is haunted by evil spirit and women eating *kochu* will suffer from constipation | 68.9 |
| Lactation | *Ongin* | Child will suffer from loose motion | 93.5 |
|  | *Marshang* | Ceases lactation | 78.4 |
| Diarrhoea | Chilli | Increases the diarhoea | 87.6 |
| Jaundice | *Apong* | Enhance the jaundice | 84.5 |
| Malaria | Bamboo shoot | Enhance malaria | 76.9 |
|  | *Onger* | Enhance diabetes | 85.3 |
| Diabetes | Tuber crops | Enhances the problem | 70.2 |

* Multiple responses of respondents
